# Unexpected Symptomatic Pneumonitis Following Breast Tangent Radiation: A Case Report

**DOI:** 10.7759/cureus.363

**Published:** 2015-10-22

**Authors:** Jessica L Conway, Karen Long, Nicolas Ploquin, Ivo A Olivotto

**Affiliations:** 1 Department of Oncology, Tom Baker Cancer Centre, Calgary; 2 Department of Medical Physics, Tom Baker Cancer Centre, Calgary

**Keywords:** breast cancer, radiation pneumonitis

## Abstract

Symptomatic radiation pneumonitis (RP) following radiation therapy (RT) to the breast alone is very uncommon. We report a case of an 80-year-old female who presented with fatigue, exertional dyspnea, fever, and cough 11.5 weeks following adjuvant breast RT with tangent fields alone. Imaging was consistent with RP, and she responded to a tapering course of steroids.

## Introduction

Radiation pneumonitis (RP) is a well-recognized toxicity following irradiation of the lung. The risk of RP following external beam RT for breast cancer varies with the volume of lung treated [1-3]. Patients typically present with a cough, dyspnea, low-grade fever, and occasionally chest pain. Pulmonary function tests may be normal. RP typically occurs within one to six months following completion of RT [2]. The MA20 trial reported clinical RP rates of 1.2% for locoregional RT (breast, axillary lymph nodes, and internal mammary chain [IMC]) and 0.2% for breast only treatment [4]. Additionally, the EORTC 22922 trial reported a higher incidence of clinical RP in patients who received locoregional RT (breast, IMCs and medial supraclavicular fields) compared to those receiving breast only treatment (0.7% vs. 0.1%, respectively) [5]. Here, we report a case of RP arising 11.5 weeks after adjuvant radiation to the breast alone.

## Case presentation

An 80-year-old female presented with a pT1c (1.3 cm) pN0 (two negative sentinel nodes), Grade 2, lobular carcinoma of the upper inner quadrant of the left breast detected incidentally on a CT scan for follow-up of colitis. Estrogen receptor status were 6/8, progesterone receptors 6/8 (both using the Allred scale), and the HER-2 gene was not over-expressed [6-7]. The lateral and posterior margins were < 1 mm.  Co-morbidities included a 25-year history of colitis not associated with inflammatory bowel disease, hypertension, hypothyroidism, and osteopenia. Our patient had a diagnostic CT scan of the chest six months before RT with no radiographic evidence of pre-existing lung disease.

Informed patient consent was obtained prior to treatment. No reference to the patient's identity is present in this paper.

**Figure 1:**
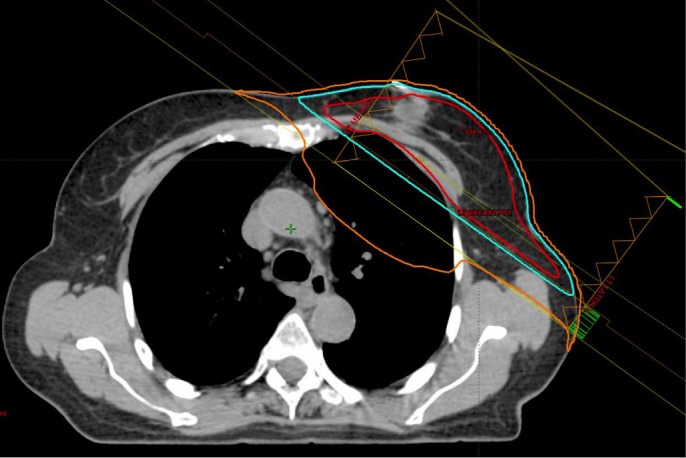
Radiation treatment planning CT scan demonstrating the 95% (red inner line), 50% (cyan middle line) and 5% (orange outer line) isodoses.

She declined endocrine therapy and was treated with RT to the left breast using a deep inspiration breath hold technique (DIBH) commencing 10 weeks after surgery. The radiation dose was 42.5 Gy in 16 fractions using a mixed 6 and 15 MV photon tangent pair, followed by a boost of 10 Gy in four fractions using 6 MeV electrons. The mean lung dose (MLD) was 4.32 Gy and the volumes of ipsilateral lung receiving ≥ 5 Gy, ≥ 20 Gy, and ≥ 30 Gy were 17%, 7%, and 6%, respectively (Figure 1). She developed brisk erythema of the skin overlying the axillary tail, but otherwise, the treatment course was uneventful. She started 3 mg of budesonide (an orally administered corticosteroid) daily for colitis seven weeks following completion of RT. She discontinued the corticosteroid after 4.5 weeks without a tapering schedule.

**Figure 2:**
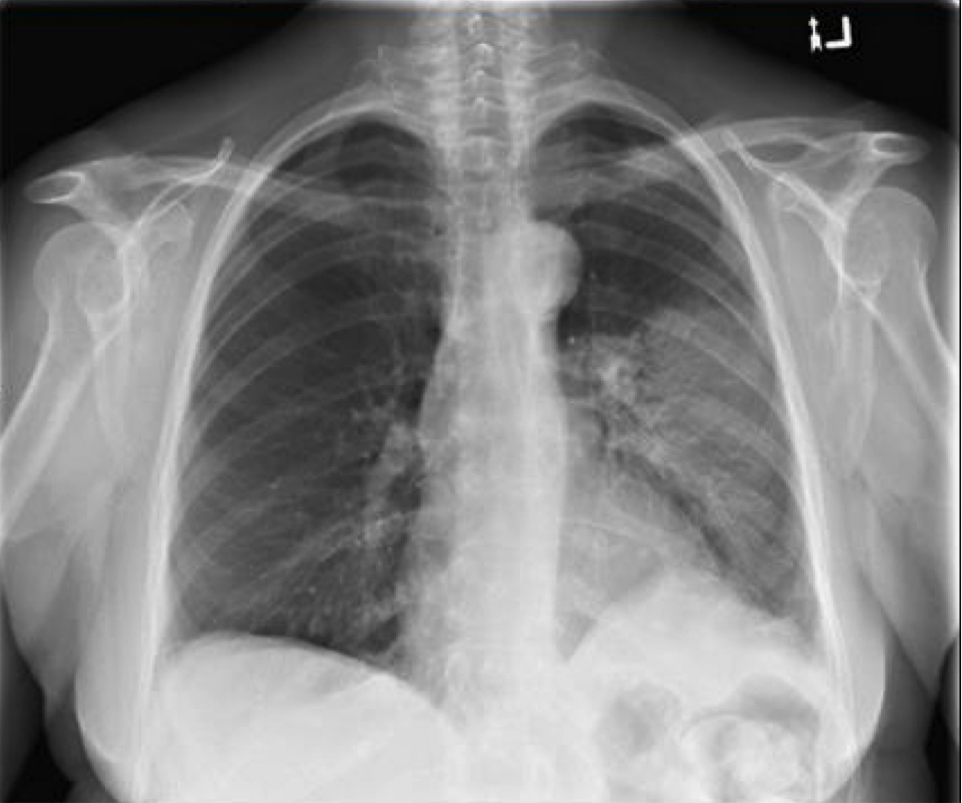
PA chest x-ray 14 weeks following completion of RT showing patchy consolidation in the central aspect of the left lung.

**Figure 3:**
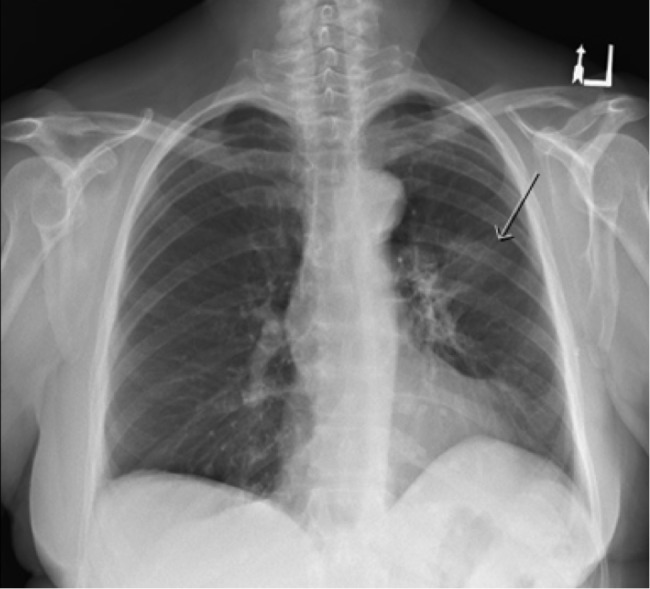
PA chest x-ray 4 weeks after starting prednisone showing significant improvement with some persistent airspace consolidation within the left upper lobe anteriorly.

Commencing 11.5 weeks after completion of RT (15 weeks from the start of RT) she noticed increasing fatigue, exertional dyspnea, fever, and cough. Two chest x-rays at 13.5 and 15.5 weeks from the completion of RT both confirmed left-sided consolidation favouring either pneumonia or a radiation reaction (Figure 2). Treatment with appropriate antibiotics failed to resolve her symptoms, which progressed over the next two weeks. On examination, moist rales were appreciated over the anterior left chest. She was not short of breath at rest. A repeat CT simulation scan using DIBH was performed in the treatment position to compare the consolidation location with the original treatment plan. The repeat CT scan was merged with the treatment planning CT, and the dosimetry and treatment fields were imported onto the new CT scan. The CT demonstrated consolidation in the anterolateral aspect of the left lung, which extended slightly above where the radiation volume projected onto the chest wall and terminated inferiorly at the lower field edge (Figure 3). 

**Figure 4:**
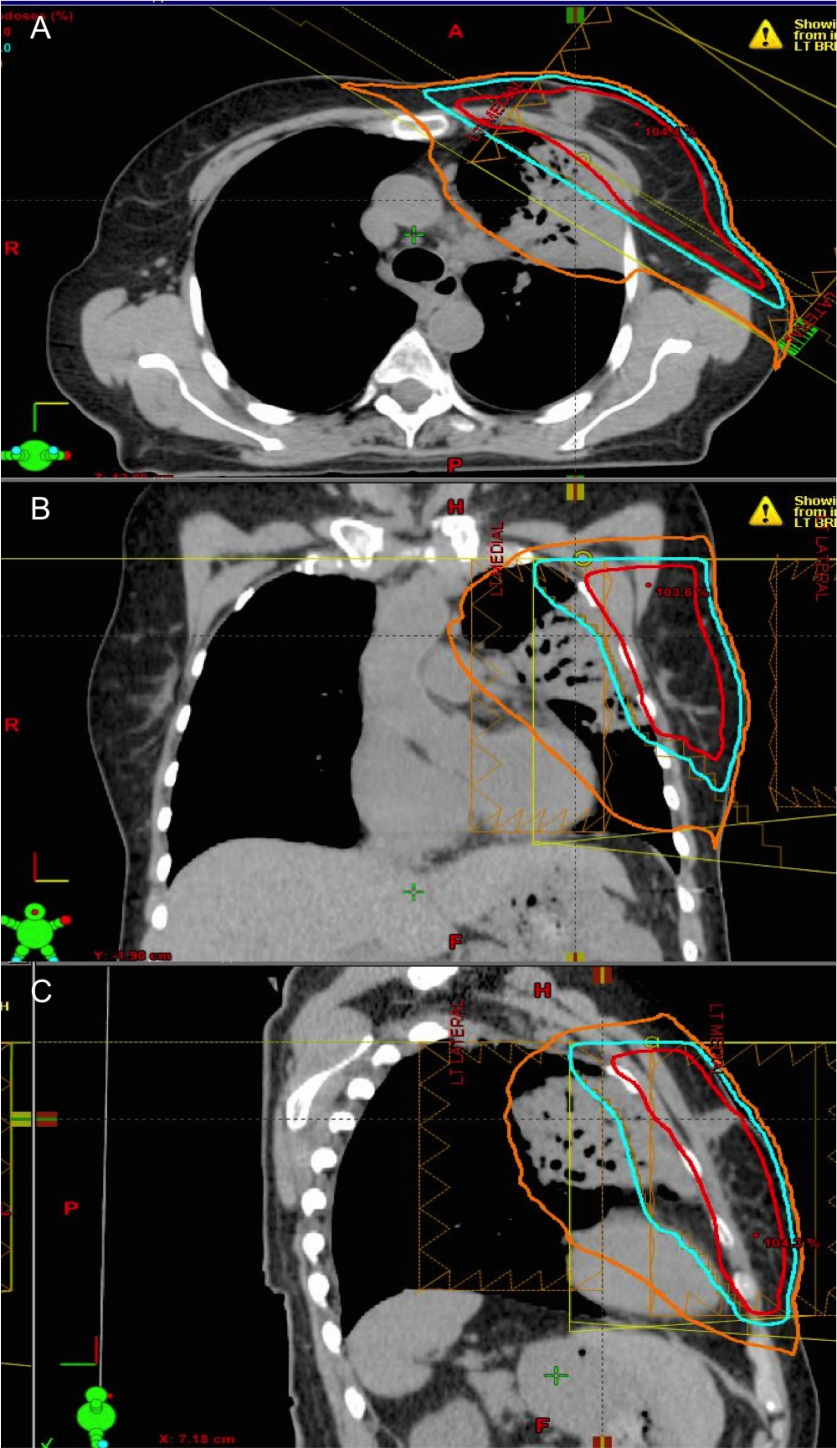
Post-RT CT simulation scan fused with the radiation treatment planning scan demonstrating consolidation. A: Axial, B: Coronal, C: Sagittal.

Two certified medical physicists independently reviewed the treatment plan and delivery parameters and confirmed that treatment delivery was as intended. The radiographic appearance, along the tangential irradiated fields and the time interval from RT, favoured RP rather than cryptogenic organizing pneumonia (COP). She started 40 mg of prednisone daily with prompt improvement in symptoms over one week. The dose of prednisone was tapered by 5 mg every six days. A chest x-ray completed four weeks after commencing prednisone showed significant improvement with some persistent airspace consolidation (Figure 4). 

## Discussion

The consequences of RP include acute symptoms, permanent lung fibrosis, and possible long-term respiratory symptoms. Cases of fatal RP have been reported [8]. RT damages small vessels and capillaries, causing vascular congestion with increased capillary permeability. The resultant inflammation promotes pulmonary capillary obstruction by platelets, fibrin, and collagen resulting in the development of permanent fibrosis [2]. Several risk factors for radiation-induced pulmonary injury have been reported. These include irradiation of the lower lobe, advancing age, pre-existing pulmonary dysfunction, specific chemotherapeutic agents, concurrent endocrine therapy, steroid withdrawal, previous RT, increasing radiation dose, increasing normal lung volume irradiated, and use of twice-daily irradiation [2-3, 9-11]. 

Generally, adverse dosimetric data, such as a higher MLD and V20, increase the risk of RP [9, 12]. Kwa, et al. reported that MLD could assist in the prediction of RP in a cohort of 540 patients, of which 59 had breast cancer [13]. However, no consistent dosimetric parameter has been identified. In our case, the ipsilateral lung V20 Gy was only 7%, a dose-volume that one would not expect to result in symptomatic RP. Furthermore, there were no radiographic characteristics of interstitial lung disease, which might have increased her risk of pulmonary toxicity. The risk of clinical RP following breast alone, tangential RT is 1-2 per thousand [4-5]. This suggests that individual genetic variation may explain the occurrence of RP in some individuals. In the lung cancer literature, an association between clinical RP and both methylenetetrahydrofolate reductase (MTHFR) genotype and ataxia telangiectasia mutated (ATM) polymorphisms has been described [14-16].

Steroid withdrawal RP has been reported in the literature resulting from a discontinuation or rapid reduction in steroid treatments in patients who have received radiation. This phenomenon has traditionally been associated with malignant lymphomas. However, there are case reports of patients treated with RT for carcinomas who developed RP following discontinuation of steroids [17]. In our patient, the abrupt discontinuation of budesonide coincided with the development of symptomatic RP. This may have exacerbated her respiratory symptoms and predisposed our patient to RP. Many patients with breast cancer receive taxane-based chemotherapy with dexamethasone support as an anti-emetic. That amount of steroid medication has not been associated with an increased risk of symptomatic RP. 

Pulmonary toxicity in patients treated with chest wall RT can occur with several different patterns, including radiation pneumonitis as described above, COP, and radiation recall pneumonitis (RPP). COP is a form of sub-acute lung damage, which typically occurs within one year after completion of RT. It is characterized by lung infiltrations extending outside of the RT fields and does not result in pulmonary fibrosis [10, 18]. COP has a higher incidence in breast cancer than other malignancies [18]. RPP develops subsequently to the use of an antineoplastic agent in patients many years following RT [10]. Our patient had consolidation of the lingula, but no overt radiographic signs to suggest COP. 

Identifying patients who are at increased risk of RP is a challenge due to the lack of consistent predictive factors and since no ideal dosimetric parameters have been identified. Improvements in radiation techniques, including the use of DIBH to reduce lung V20, may help decrease the risk of RP [19-20]. However, in our case, despite advanced technology, the use of DIBH, and excellent lung dosimetric parameters, the occurrence of symptomatic RP was not prevented. This suggests an idiosyncratic reaction or unknown factors contributed to the development of RP.

## Conclusions

Our report describes a case of symptomatic RP occurring 11.5 weeks following adjuvant radiation to the breast with tangents alone. Dosimetric parameters included an ipsilateral lung V20 of only 7% and a MLD of 4.32 Gy. Although RP is uncommon following breast alone RT, it is a real risk and should be discussed with patients. 
